# *SoxC* is Required for Ecdysteroid Induction of Neuropeptide Genes During Insect Eclosion

**DOI:** 10.3389/fgene.2022.942884

**Published:** 2022-07-11

**Authors:** Guang-Hua Luo, Xi-En Chen, Yao-Yu Jiao, Guan-Heng Zhu, Ru Zhang, Ramesh Kumar Dhandapani, Ji-Chao Fang, Subba Reddy Palli

**Affiliations:** ^1^ Institute of Plant Protection, Jiangsu Academy of Agricultural Sciences, Jiangsu Key Laboratory for Food and Safety-State Key Laboratory Cultivation Base of Ministry of Science and Technology, Nanjing, China; ^2^ Department of Entomology, College of Agriculture, Food and Environment, University of Kentucky, Lexington, KY, United States; ^3^ School of Agriculture, Sun Yat-sen University, Shenzhen, China

**Keywords:** SoxC, 20E, ecdysis behavior, EH, metamorphosis

## Abstract

In insects, the shedding of the old exoskeleton is accomplished through ecdysis which is typically followed by the expansion and tanning of the new cuticle. Four neuropeptides, eclosion hormone (EH), ecdysis triggering hormone (ETH), crustacean cardioactive peptide (CCAP) and bursicon (Bur) are known to control ecdysis. However, the regulation of these neuropeptide genes is still poorly understood. Here, we report that in the red flour beetle (RFB) *Tribolium castaneum* and the fall armyworm (FAW) *Spodoptera frugiperda*, knockdown or knockout of the *SoxC* gene caused eclosion defects. The expansion and tanning of wings were not complete. In both RFB and FAW, the knockdown or knockout of *SoxC* resulted in a decrease in the expression of *EH* gene. Electrophoretic mobility shift assays revealed that the SfSoxC protein directly binds to a motif present in the promoter of *SfEH*. The luciferase reporter assays in Sf9 cells confirmed these results. These data suggest that transcription factor *SoxC* plays a key role in ecdysteroid induction of genes coding for neuropeptides such as EH involved in the regulation of insect eclosion.

## Introduction

Insects accommodate new growth by periodic shedding of their cuticle (exoskeleton) through an innate ecdysis behavior (Ewer, 2005; Nassel and Zandawala, 2019). During ecdysis, the insect performs a series of repetitive movements, known as an ecdysis behavioral sequence. The ecdysis sequence is composed of pre-ecdysis, ecdysis and post-ecdysis behaviors, which are controlled by four critical neuropeptides: Eclosion hormone (EH), ecdysis triggering hormone (ETH) or ecdysis triggering hormone precursor (ETHp), crustacean cardioactive peptide (CCAP) and bursicon (Bur) (White and Ewer, 2014). The initial trigger of the ecdysis behavioral sequence is the release of ecdysteroid (20-Hydroxyecdysone, 20E is the most active form)-dependent ETHp from the epitracheal Inka cells. ETH subsequently induces the release of EH from brain neurons, through a positive feedback loop between ETH and EH; both neuropeptides are then released (Chang et al., 2009). Following the rise of ETH, the accompanying release of EH within the central nervous system (CNS) causes the release of CCAP. CCAP then turns on the ecdysial motor program. Following ecdysis, the post-ecdysial phase is regulated by the secretion of bursicon from a subset of CCAP-expressing neurons in the abdominal ganglia (Truman, 2005). During the post-ecdysis phase, processes such as wing expansion, cuticle tanning and hardening occur (Arakane et al., 2008; Peabody et al., 2008; Flaven-Pouchon et al., 2020). Previous studies showed that the expression of neuropeptide genes mentioned above are induced by 20E (Chang et al., 2009; Bai and Palli, 2010; Li et al., 2020). However, the mechanisms of action of 20E in the induction of neuropeptide genes are not well understood.

The ecdysis sequential model proposes that four neuropeptides involved in regulation of ecdysis are released in a sequential manner and act t independently to to ecdysis behavioral. However, some studies suggest that different neuropeptides may exert overlapping and multiple functions rather than strictly sequential actions (White and Ewer, 2014). In *Drosophila melanogaster*, the ﬂies lacking both EH and CCAP-expressing neurons have more severe ecdysis defects than those lacking either one (Clark et al., 2004). The flies in which the CCAP-expressing neurons are genetically ablated showed only minor defects during larval ecdysis, but showed severe defects during pupal ecdysis (Park et al., 2003). Besides, the mechanisms that regulate ecdysis sequences may vary across species, with the same neuropeptide playing different roles in different insects. For example, ETHp regulates only pre-ecdysis in *Manduca sexta,* whereas in *Bombyx mori* it can trigger the entire ecdysis sequence (Zitnan et al., 2002).

*Sox* genes, characterized by the conserved high mobility group (HMG) box, encode a class of transcription factors with high sequence similarity to the sex-determining region of Y chromosome (*SRY*) gene in animals (Yu et al., 2018). The transcription factor comprises a group of proteins characterized by the presence of an SRY box, thus named SOX (Pevny and Lovell-Badge, 1997). Till now, more than 100 *sox* genes have been discovered in animals, including insects (Sinclair et al., 1990). The Sox genes are classified into 12 subfamilies (A, B1, B2, C, D, E, F, G, H, I, J, and K) (Yu et al., 2018). Eight *sox* genes have been identified in *D. melanogaster* (Crémazy et al., 2001; Yu et al., 2018). *Sox14* (which belongs to *SoxC* subfamily) is a primary response gene induced by 20E (Beckstead et al., 2005). In *l(2)mbn* cells, the *Sox14* expression alone is sufficient to induce apoptosis (Chittaranjan et al., 2009). Besides, *Sox14* is required for the complete destruction of the larval midgut and is a positive regulator of salivary gland cell death in *D. melanogaster*. The *Sox14*-RNAi flies revealed phenotypes characteristic of aberrant or reduced ecdysone signaling, including defects in larval midgut and salivary gland destruction (Beckstead et al., 2005; Chittaranjan et al., 2009).

In the fall armyworm, *Spodoptera frugiperda*, showed that the expression of *SoxC* increased at pre-molting stage and reached the maximum levels in the newly ecdysed pupae and adults. The same expression patterns were also observed in the red flour beetle, *Tribolium castaneum*. These data suggest that the *SoxC* plays an important role in regulating the eclosion behavioral sequence, therefore, we studied the function of *SoxC* gene in insect eclosion.

## Materials and Methods

### Insect Strains

The wild-type GA-1 strain of the red flour beetles, *T. castaneum*, were reared on organic wheat flour containing 10% yeast at 30°C. The final instar larvae were staged based on untanned white head phenotypes observed immediately after molting.

The fall armyworm, *S. frugiperda*, larvae were reared on artificial diet at 27 ± 1°C, 14 h:10 h (light: dark) photoperiod and 65 ± 5% relative humidity (RH). Each larva was placed in a separate cup. Pupae were sexed and kept in cages for adult eclosion. Pupae were checked daily for emergence and the moths were fed on 10% sugar water.

### Identification of *SoxC* Genes

The gene encoding SoxC transcription factor from *T. castaneum* and *S. frugiperda* were identified by searching the non-redundant protein sequences (NR) database at NCBI using *D. melanogaster SoxC* gene (Flybase ID: FBgn0005612) protein sequence as a query. Candidate sequences for the *SoxC* gene in *T. castaneum* (XP_973116.1) and *S. frugiperda* (XP_035435714.1) were amplified by reverse transcription PCR (RT-PCR) using primers shown in Supplementary Table S1.

### RNA Extraction, cDNA Synthesis and RT-qPCR Analysis

Total RNA was isolated from treated and control insects using TRIzol reagent (Thermo Fisher Scientiﬁc). The concentration of RNA was measured using a NanoDrop-2000 spectrophotometer (Thermo Fisher Scientiﬁc). cDNA was synthesized from 2 µg of total RNA samples using M-MLV reverse transcriptase (Invitrogen, Thermo Fisher Scientific) in a 20 µL reaction following the manufacturer’s protocol. Diluted (10×) cDNA, gene-specific primers and iTaq Universal SYBR Green Supermix (Bio-Rad, Hercules, CA) were used in reverse transcriptase quantitative PCR (RT-qPCR). All the primers used for RT-qPCR are shown in Supplementary Table S1.

### Double-Stranded RNA (dsRNA) Synthesis

To prepare the dsRNA of *TcSoxC* gene, a pair of specific primers with T7 polymerase promoter (5′-TAA​TAC​GAC​TCA​CTA​TAG​GG-3′) at their 5′-end were used to amplify a 437 bp fragment of the *TcSoxC* gene (Supplementary Table S1). The PCR products were purified using QIAquick PCR purification kit (QIAGEN, Hilden, Germany), which were then used as templates for dsRNA synthesis using the Invitrogen 5× MEGAscript T7 Transcription kit (Invitrogen, Thermo Fisher Scientific Inc., Vilnius, Lithuania). The dsRNA was treated with DNase I (Ambion, Austin, TX) and puriﬁed using a phenol/chloroform extraction followed by ethanol precipitation. Finally, the dsRNA was dissolved in nuclease-free water and checked for quality by electrophoresis on agarose gel. The concentration of dsRNA was measured using a NanoDrop-2000 spectrophotometer (Thermo Fisher Scientiﬁc Inc., Waltham, MA) to be 3–5 μg/μL. The control dsRNA targets the *malE* gene of *Escherichia coli* (Bai and Palli, 2010). All dsRNA products were stored in −20°C.

### Synthesis of sgRNAs Targeting *SfSoxC*

sgRNAs were designed based on the criteria: 5′-GG-(N)18-NGG-3'. sgRNA templates were produced (Supplementary Table S1) as described previously (Zhu et al., 2016; Zhu et al., 2020) using the MAXIscript T7 *In Vitro* Transcription Kit (Invitrogen) following the manufacturer’s instructions. The purified sgRNAs were quantified using a NanoDrop-2000 spectrophotometer (Thermo Fisher Scientiﬁc) and stored in −70°C. The Cas9 protein used in our study was purchased from Thermo Fisher Scientiﬁc (TrueCut Cas9 Protein v2, A36497, Invitrogen).

### dsRNA Microinjection Into *T. castaneum*

Newly molted final instar larvae were used for microinjection. 400 ng of dsRNA was injected into each larva on the dorsal side of the abdomen. The NANOJECT III (Drummond Scientific Co.) was used for dsRNA microinjection with an aspirator tube assembly ﬁtted with 3.5″ glass capillary tube (Drummond Scientific Co.) pulled by a needle puller (Model P-1000, Sutter Instruments Co., Novato, CA). Injected larvae were allowed to recover for 1 h at room temperature and then transferred to a 30°C incubator under standard conditions. Control larvae were injected with dsRNA targeting *E. coli malE* gene.

### sgRNA and Cas9 Protein Microinjection Into *S. frugiperda* Embryos

The *S. frugiperda* adults were placed in a cage (L × W × H: 15 cm × 15 cm × 10 cm) that was covered with brown paper on the top and fed with 10% sugar water. The fresh eggs (laid within 1 h) were lined on a microscopic slide, and a mixture containing 300 ng/μL Cas9 protein (TrueCut Cas9 Protein v2, A36497, Invitrogen) and 100 ng/μL of each sgRNA was injected into less than 1 h old eggs using a microinjector (NARISHIGE IM300, Japan). To increase mutation efficiency, three sgRNAs of the same concentration were co-injected (Zhu et al., 2020). Injected eggs were kept in a sterile petri dish at 27 ± 1°C until hatching.

### Mutagenesis Analysis in *S. frugiperda*

The mutagenesis was assessed using gene fragment sequencing and T7EI assay. Genomic DNA was isolated from individual insects using DNeasy Blood & Tissue Kit (QIAGEN) following the manufacturer’s protocol. PCR was carried out using the PrimeSTAR GXL DNA Polymerase (TaKaRa) in 50 μL reaction to amplify the target fragments from the genomic DNAs. PCR products was purified using QIAquick PCR purification kit (QIAGEN) and were cloned into TOPO PCR4 vector (Invitrogen, Thermo Fisher Scientiﬁc) following the manufacturer’s protocol. 10G competent cells were then transfected with the plasmid construct and positive clones were sequenced. Primers are shown in Supplementary Table S1.

To estimate the mutation rate, the T7EI assay was performed using the PCR products as described previously (Zhu et al., 2020). Briefly, a total 200 ng of PCR products in 1 × NEB buffer 2 were hybridized under the following cycles: 95°C for 5 min, 95–85°C at -2°C ^s−1^, 85–25°C at −1°C ^s−1^, and held at 4°C. Then, 10 units of T7EI (New England Biolabs) was added to the reaction and incubated at 37°C for 15 min. Finally, the reaction mixtures were run on 2% agarose gels containing Gel Red, and the gel electrophoresis results were photographed under a UV light. The image lab (Bio-lab) software was used for analyzing gel images. The percentage of mutagenesis was calculated using the method described previously (Zhu et al., 2020).

### Effects of 20E on the Expression of *TcSoxC in vivo*

Technical grade stable ecdysone agonist (SEA), RH-102240 [N-(1,1-dimethylethyl)-N'-(2-ethyl-3-methoxybenzoyl)-3,5-dimethylbenzohydrazide] was dissolved to 100 μg/μL in acetone. Newly molted final instar larvae of *T. castaneum* were injected with 400 ng of *TcSoxC* dsRNA. When the injected individuals entered into quiescent stage (q-stage) and the compound eyes appeared, 2 µL of SEA solution was added on the dorsal side abdomen of the insects. The control insects were treated with the same volume of acetone. 12 h after the treatment, insects were sampled and total RNA was isolated. The expression of target genes was determined using RT-qPCR and the primers used are listed in Supplementary Table S1.

### Phenotype Photographs

Phenotypes of both *T. castaneum* and *S. frugiperda* were observed during eclosion (pupal-adult metamorphosis). All the phenotypes were photographed using a Nikon stereomicroscope (SMZ745T, Nikon, Japan). Eclosion behavior of *T. castaneum* after *TcSoxC* RNAi was recorded at 28 ± 1°C using a Canon digital camera VIXIA HF G50 video recorder.

### *SoxC* Binding Motif Analysis

To determine if SfSoxC protein directly binds to the *SfEH* promoter, we performed electrophoretic mobility shift assay (EMSA) using a probe targeting the putative *SoxC*-binding motifs located within the *SfEH* promoter region. EMSA was performed using the LightShife Chemiluminescent EMSA Kit (Thermo Fisher Scientiﬁc) following the manufacturer’s protocol. All the primers used for probes are shown in Supplementary Table S1.

Using the PCR-based accurate synthesis (PAS) method, the *SfSoxC* gene ORF was cloned into the prokaryotic expression vector pCzn1 between the *NdeI* and the *XbaI* sites (Zoonbio Biotechnology, China). The recombinant plasmid pCzn1-SfSOXC was transformed into TOP10 *E. coli* strain for sequencing. After nucleotide sequence was determined, the recombinant plasmid pCzn1-SfSOXC was transformed into the Arctic-Express (DE3) *E*. *coli* strain (Zoonbio Biotechnology, China) for recombinant protein expression. Expression of the target gene was induced using IPTG (final concentration 0.5 mM). The recombinant histidine-tagged His-SfSOXC protein was detected in the supernatant and purified by affinity chromatography using a Ni-IDA-Sepharose Cl-6B Microspin column (Zoonbio Biotechnology, China). The recombinant protein was confirmed by western blot analysis.

### Luciferase Activity Assay

To construct promoter-pG5luc vectors for the luciferase assay, the potential promoter sequences of *SfEH*, containing 20 bp homologous arms for the pG5luc vector digested with *KpnI* and *HindIII*, were amplified from genomic DNA using Prime STAR GXL DNA Polymerase (TakaRa). The amplified products were inserted into pG5luc vector using the Gibson assembly master mix (NEB) to yield the *SfEH/P2574-pG5luc* vector. The ORF of *SfSoxC* genes, containing 20 bp homologous arms for the pIEx-4 vector digested with *NcoI* and *AscI*, were amplified using Prime STAR GXL DNA Polymerase (TakaRa), and then cloned into *NcoI* and *AscI* digested pIEx-4 vector as described above, yielding *pIEx-4-SfSoxC* vector.

To determine whether *SfSoxC* activates *SfEH* promoter, luciferase assays were carried out in the Sf9 cells. Sf9 cells were seeded in 96-well culture plates at a density of 2 × 10^5^ cells per well and incubated at 27°C overnight. The cells were then transfected with 100 ng of constructs per well using 0.8 μL of CellFectin II reagent (Thermo Fisher Scientiﬁc) in 50 μL of Sf-900 II medium. At 4 h post-transfection, the medium was removed and replaced with 100 μL fresh medium. Luminescence was quantified using SpectraMax i3x (San Jose, CA) at 48 h post-transfection. Medium was removed and cells were washed with 100 μL of 1 × PBS, then 100 μL of ice-cold lysis buffer was added to each well. The cell plates were placed on a shaker for 10 min at room temperature. 20 and 10 μL of cell lysate were used for luciferase activity assay and protein concentration determination, respectively, as described previously (Chen et al., 2020).

### Statistical Analysis

Results are presented as means ± SE. Statistical analyses were conducted in SPSS version 24. Treatments were compared using unpaired *t-test* with variances treated separately. All tests were conducted by one-tailed test.

## Results

### 20E Induction of Neuropeptide Genes Require *SoxC*

**FIGURE 1:**
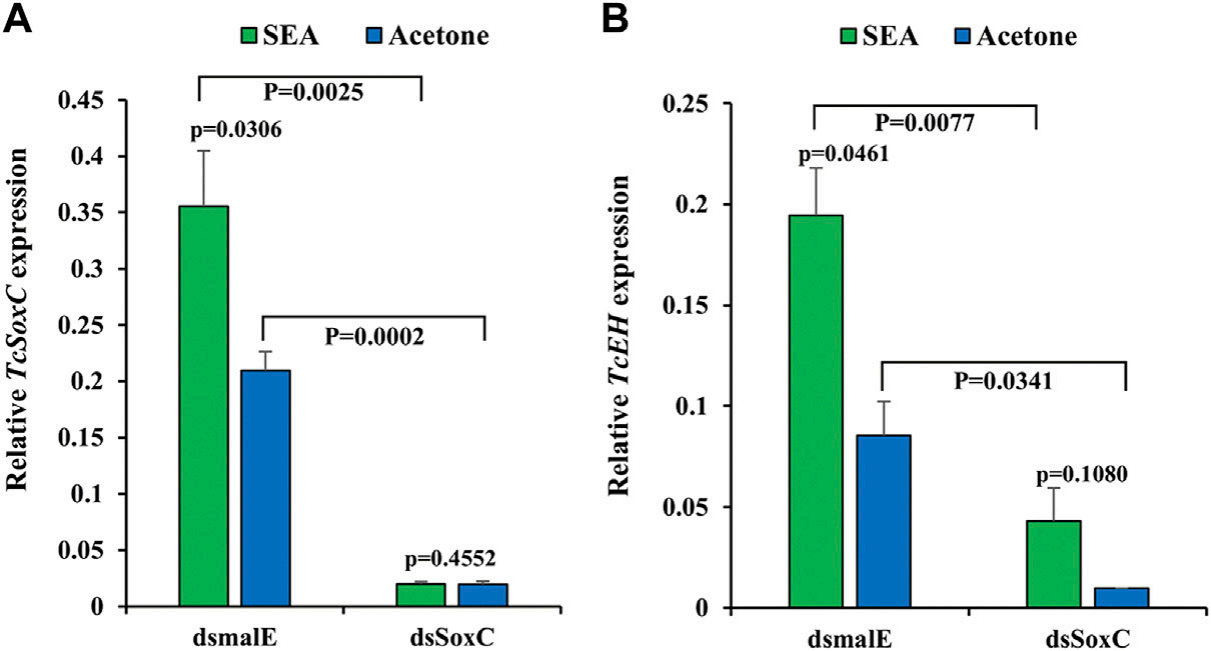
*SoxC* is required for 20E induction of *EH* in *T. castaneum*. Newly molted final instar larvae were injected with 400 ng of ds*SoxC* and 2 µL of SEA solution (dissolved in acetone) was added on the dorsal side abdomen of insects upon entry into quiescent stage and emergence of compound eyes. The control group was treated with acetone. 12 h after treatment, insect total RNA was isolated and quantified with RT-qPCR. **(A)** The expression of *TcSoxC* increased significantly in larvae from the control group treated with SEA, but this increase was blocked larvae treated with both ds*SoxC* and SEA. **(B)** The expression of *TcEH* increased significantly in larvae from the control group treated with SEA but this increase was blocked larvae treated with both ds*SoxC* and SEA. Means ± SE of three independent experiments are shown (*p* values come from *t tests*).

In the red flour beetle, after stable ecdysone agonist (SEA) treatment, *TcSoxC*, *TcEH*, *TcCCAP* and *TcBur* mRNA levels increased compared to those in control insects treated with solvent. Injection of ds*SoxC* before treatment with SEA reduced induction of *EH, CCAP* and *Bur* genes by SEA (Figure 1; Supplementary Figure S1). These data suggest that SoxC may be required for ecdysone regulation of the neuropeptide genes tested.

### Phenotypes of *TcSoxC* Knockdown in *T. castaneum*

**FIGURE 2:**
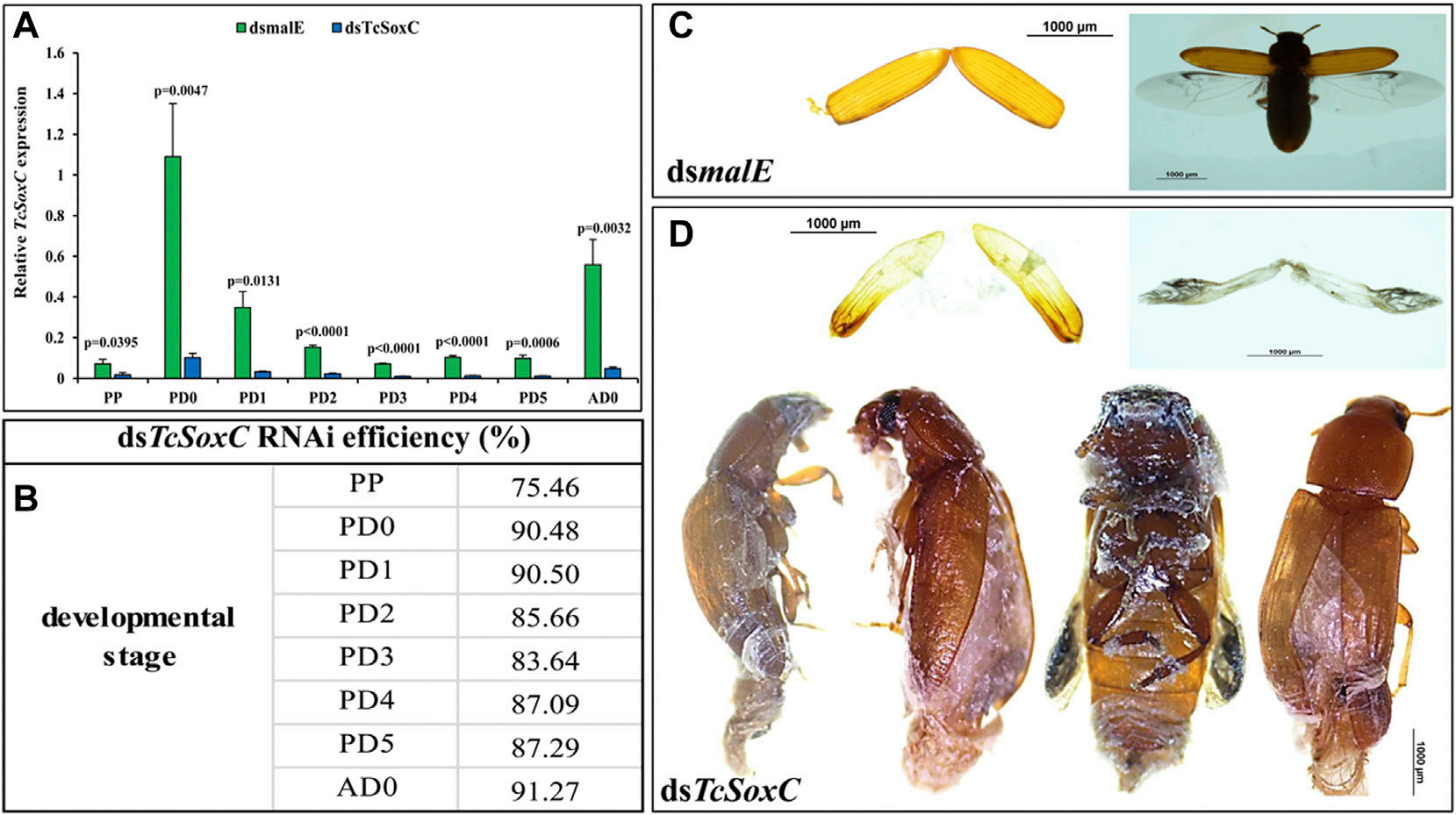
RNAi efficiency and phenotypes of *TcSoxC* knockdown in *T. castaneum*. 400 ng of ds*SoxC* was injected into each larva on the dorsal side of the abdomen. Control larvae were injected with ds*RNA* targeting *E. coli malE* gene. **(A)** The expression of *TcSoxC* decreased significantly after *TcSoxC* knockdown at different developmental stages. **(B)** The RNAi efficiency of *Tc-SoxC* knockdown ranged from 75.46% (PP) to 91.27% (AD0). **(C)** The phenotypes of ds*malE* injected insects. **(D)** The phenotypes of ds*SoxC* injected insects. The tanning as well as the expansion of forewings (elytra) and hindwings (membranous wings) were abnormal in ds*SoxC* treated insects. Four randomly selected insects were injected with ds*TcSoxC*, leading to incomplete molting and shedding of the pupal cuticle. Means ± SE of three independent experiments are shown (*p* values comes from *t tests*). PP: pre-pupa, PD0: newly molted pupa (0 h after ecdysis into the pupal stage), PD1: 24 h after ecdysis into the pupal stage, PD2: 48 h after ecdysis into the pupal stage, PD3: 72 h after ecdysis into the pupal stage, PD4: 96 h after ecdysis into the pupal stage, PD5: 120 h after ecdysis into the pupal stage, AD0: newly eclosed adult. (For interpretations of color reference in this figure legend, the reader is referred to the Web version of this article.)

Injection of ds*SoxC* into newly molted last instar larvae triggered 75–91% knockdown of target gene in pupae and adults (Figures 2A,B). The ds*SoxC*-treated larvae progressed to the pupal stage but were unable to complete the transition from pupa to adult. Some of the pharate adults were stuck inside pupal integument. The tanning, as well as the expansion of the forewings (elytra) and hindwings, were abnormal in ds*SoxC* treated insects (Figure 2D) compared to those treated with control *dsmalE* (Figure 2C).

As expected, the expression of neuropeptide genes (*EH*, *CCAP* and *Bur*) decreased in ds*SoxC* treated insects compared to that in control insects treated with ds*malE* (Supplementary Figure S2). Although the expression of *ETHp* gene also decreased in ds*SoxC* treated insects, the decrease in expression is not consistent in all stages tested. For example, the expression of *ETHp* increased in day 4 pupae in ds*SoxC*-treated larvae compared to the control insects (Supplementary Figure S2B).

### Phenotypes of *SfSoxC* Knockout in *S. frugiperda*

**FIGURE 3:**
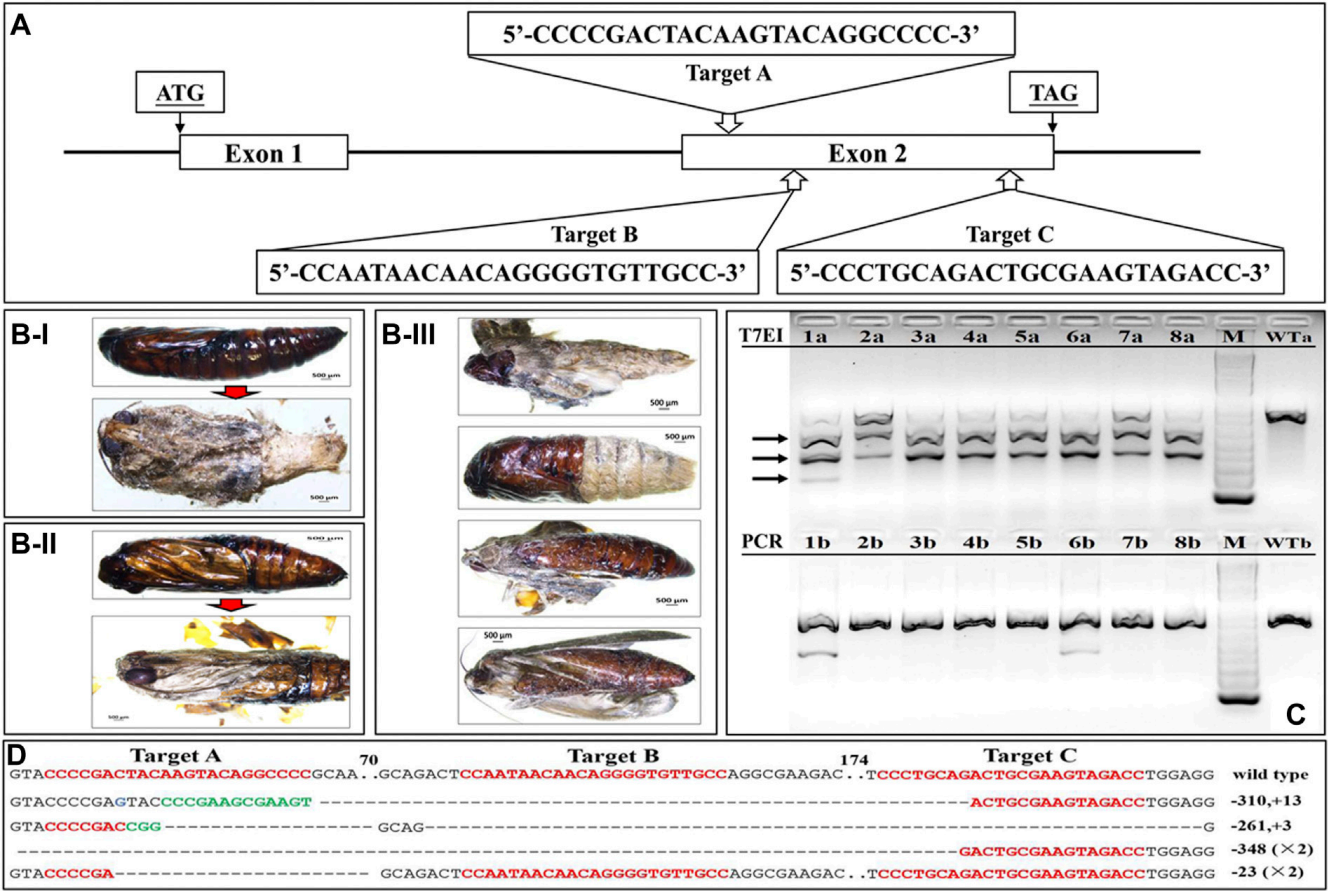
Site-specific mutagenesis and phenotypes of *SfSoxC* knockout in *S. frugiperda*. Fresh eggs (laid within 1 h) were lined on a microscopic slide, and a mixture containing 300 ng/μL Cas9 protein and 100 ng/μL of each sgRNA was injected into eggs that were less than 2 h old. Three sgRNAs were co-injected together with the same concentration. Injected eggs were kept in a sterile petri dish at 27 ± 1°C until hatching. **(A)** Schema of sgRNA target site in exon2 of *SfSoxC* gene. Three target sites (Target A, Target B, Target C) were selected for genome editing. ATG is the translation initiation codon and TAG is the translation termination codon. **(B)** The phenotypes of *SfSoxC* knockout. Inside the pupa, compound eyes, antennae, mouthparts, and appendages were clearly seen but the wings were poorly developed **(B-I and B-II)**. The pupal shell was not shed from body completely **(B-III)**. **(C)** The mutagenesis in *SfSoxC* gene confirmed by T7EI assay. Genomic DNA was isolated and PCR-amplified from 8 randomly selected adults (1–8) that showed *SfSoxC* gene knockout phenotype. The amplified products were run on agarose gels with (T7EI, 1a-8a) or without (PCR, 1b-8b) T7EI endonuclease digestion. The arrows on the left show the digested products. The DNA marker was 1 kb ladder (M). The wildtype controls are shown (WTa and WTb). **(D)** Mutant alleles identified by TA clone sequencing. Wild-type sequences are shown at the top with the 3 target sites marked by red letters. Deletions are shown as dashes and insertions are shown as green letters. The net change in length is shown at the right of each sequence (+: insertion; −: deletion). ×2 on the right side of a sequence denote that this mutant type was detected in two sequenced clones. Numbers in the figure represent the number of base-pairs. (For interpretation of the references to color in this figure legend, the reader is referred to the Web version of this article.)

Three sgRNAs targeting exon2 of *SfSoxC* were used to knockout *SoxC* gene in *S. frugiperda* (Figure 3A). A total of 216 eggs were injected with Cas9 protein and sgRNAs (Supplementary Table S2). 83% mutation rate was detected in 30 larvae tested. Some of the injected larvae (39) died during first instar larval stage (Supplementary Table S2). Thirty-nine larvae developed to pupal stage and died at the end of the pupal stage (Figures 3B-I,II). Forty-eight insects showed abnormal phenotypes during eclosion (Figure 3B-III; Supplementary Table S2). The pharate adults were stuck inside pupal cuticle and the expansion of wings was not complete (Figure 3B-III). To confirm the mutagenesis in *SfSoxC* gene induced by CRISPR/Cas9, T7EI assay was performed. PCR amplification of mutated sequences was carried out using genomic DNA isolated from eight randomly selected mutants. The mutant DNA was cut into three fragments by T7EI (Figure 3C-top) and only one band without T7EI treatment (Figure 3C-bottom). Furthermore, to determine the indels of *SfSoxC* gene, the PCR products were cloned and sequenced. Four different alleles displaying mutations in 15 sequenced clones were identified (Figure 3D). According to the T7EI assay, the heterozygotes develop an abnormal eclosion molt phenotype and there were no individuals for doing a backcross. Besides, some homozygotes died at 1^st^ instar larval and the others develop an abnormal eclosion molt phenotype (Figure 3B-III; Supplementary Table S1).

Expression of the four neuropeptide genes significantly changed after disrupting the *SfSoxC* gene (Supplementary Figure S3). At all four stages checked, *EH* and *Bur* expression decreased significantly (Supplementary Figures S3A,D), whereas the expression of *ETHp* increased significantly (Supplementary Figure S3B). The expression of *CCAP* gene slightly decreased at PP and PD0, and decreased significantly at PD7 and AD0 stages (Supplementary Figure S3C).

**FIGURE 4:**
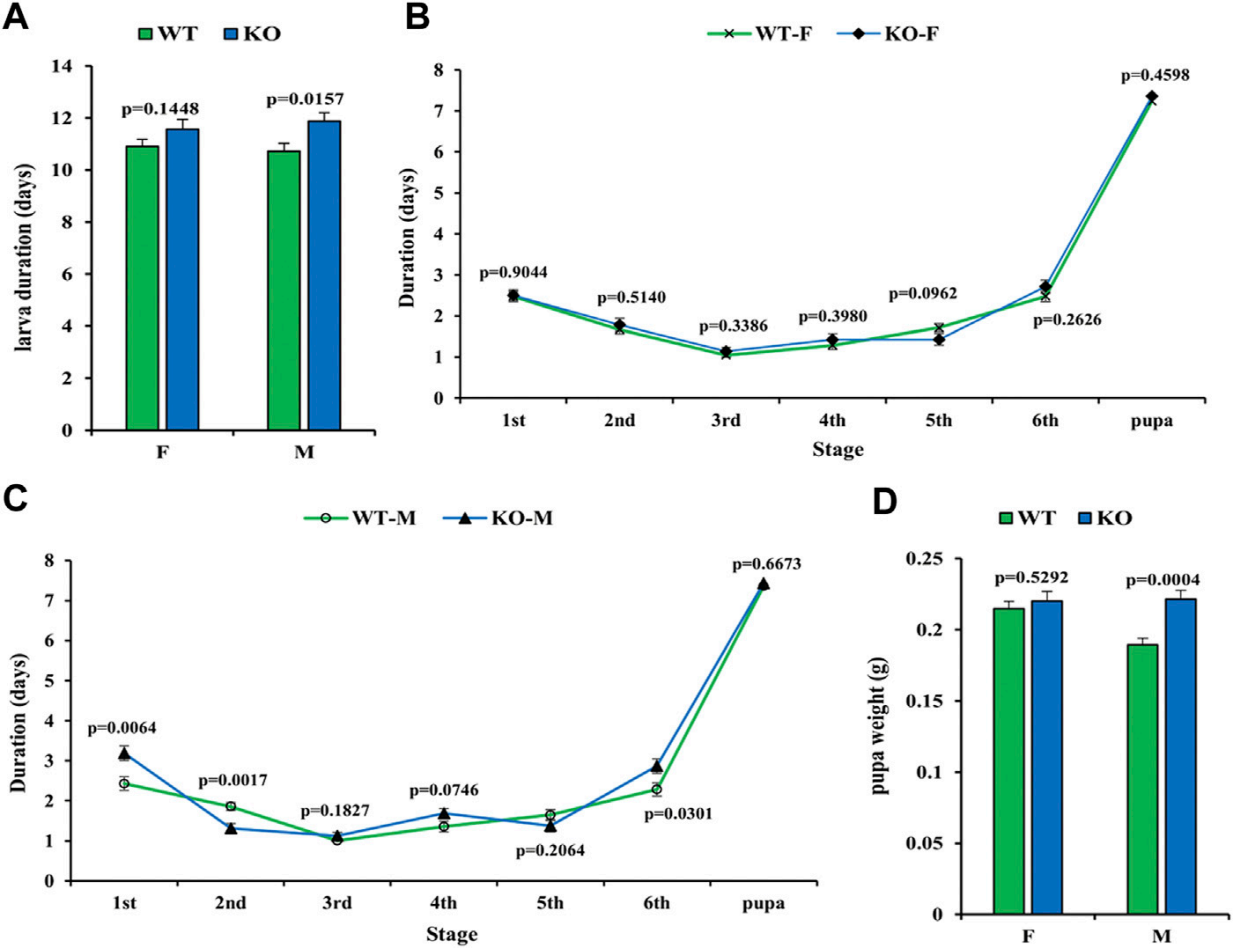
Effect of *SfSoxC* knockout on the larval development time and pupal weight. The developmental time and pupal weight were determined in *SfSoxC* knockout fall armyworm. **(A)** The larval development time (days) between wildtype (WT) and *SfSoxC* gene knockout (KO) insects showed no significant difference (*p* = 0.1448) in female **(F)**, but showed significant difference (*p* = 0.0157) in male (M). **(B)** The developmental time (days) at each developmental stage in female (WT-F and KO-F) insects. There is no significant difference between WT and KO insects at any developmental stage. **(C)** The development time (days) at each developmental stage in male (WT-M and KO-M) insects. There is no significant difference between WT and KO insects at 3rd, 4th, 5th and pupa stages. During the 1^st^ and 6^th^ stages, however, the developmental time of the KO-M insects is significantly longer than that of the WT-M insects. During the 2^nd^ instar, the developmental time of the KO-M insects is significantly shorter than that of the WT-M insects. **(D)** The pupal weight (g) between WT and KO insects has no significant difference (*p* = 0.5292) in female (F), but showed significant difference (*p* = 0.0004) in male (M). Means ± SE of three independent experiments are shown (*p* values comes from *t tests*).

To test whether the development of insects is affected by *SfSoxC* gene knockout, the development time and pupa weight were recorded. In females, neither the development time nor pupal weight showed any significant difference between wild-type and mutants (Figures 4A,B,D). However, in males, significant differences in development time of 1st, 2nd, and 6th instar larvae and pupal weight were observed (Figures 4A,C,D).

### *SfSoxC* Gene Directly Binds to the Promoter of *SfEH*

**FIGURE 5:**
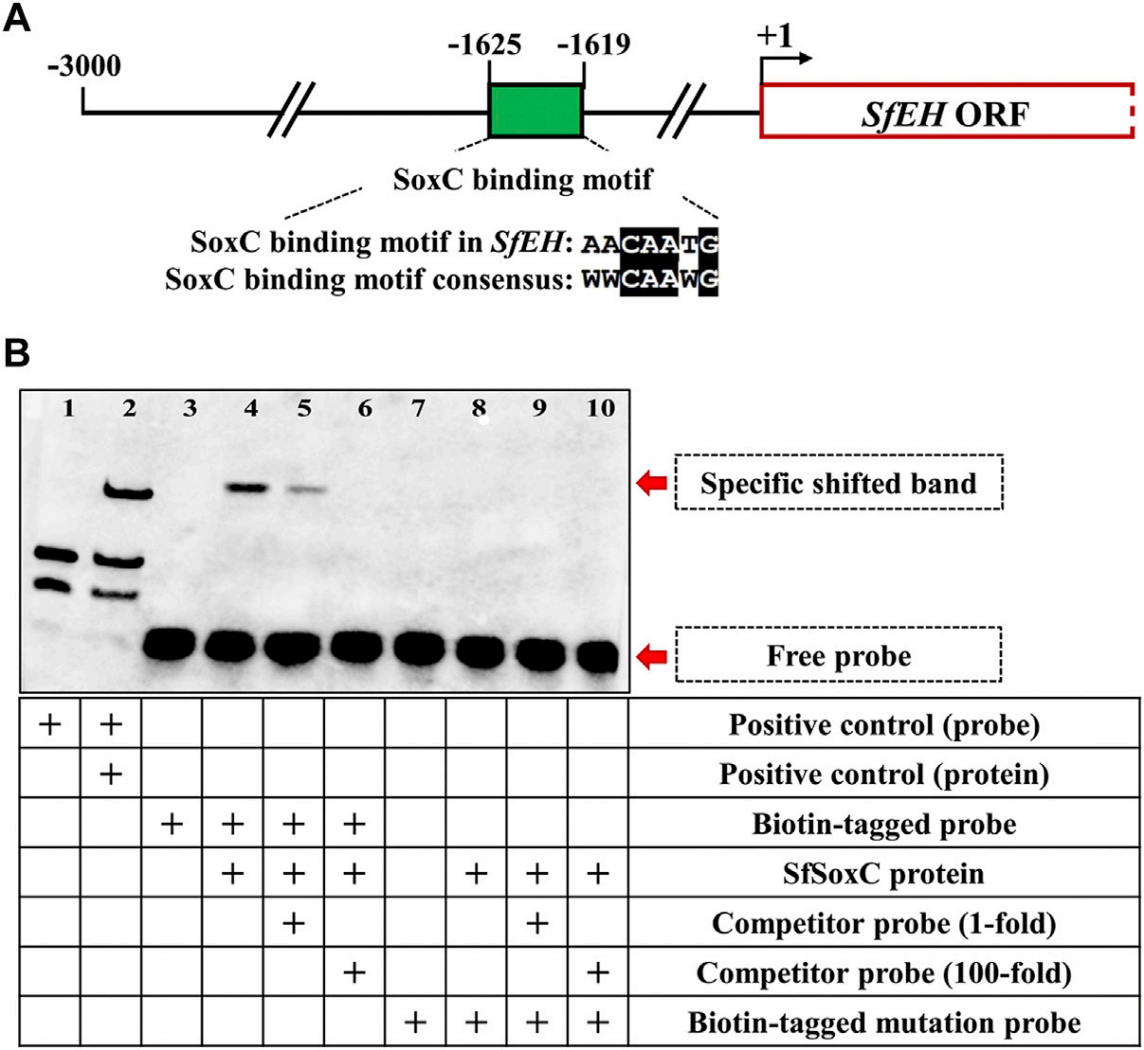
The *SfSoxC* directly binds to the promoter of *SfEH*. The binding motif of *SfSoxC* identified in the promoter of *SfEH* was confirmed by EMSA. **(A)** The predicted Sf-SoxC-binding motif (5′-AACAATG-3′) in the *SfEH* promoter region. The numbers indicate the distance from the start codon (+1) of *SfEH* gene. The putative SfSoxC-binding motif in *SfEH* promoter region is aligned with the motif consensus sequence (5′-WWCAAWG-3′). **(B)** Lane 1 and 2 is the positive control. Lane 3 is the biotin-tagged probe loading only. Lane 4, there is a specific shift band when SfSoxC protein and biotin-tagged probe were incubated together. The competition assays showed that the specific band became weak with the addition of a 1-fold molar excess of unlabeled probe (competitor probe) and completely disappeared with the addition of a 100-fold molar excess of unlabeled probe (lanes 5 and 6, respectively). There is no specific band shifting when biotin-tagged mutation probe was incubated with SfSoxC protein (lane 8, 9 and 10).

To determine if *SfSoxC* directly binds to the conserved SoxC binding motif in the promoter of *SfEH*, we performed EMSA using a probe containing the SoxC-binding motif sequences found in the promoter of *SfEH* (Figure 5A). A specific shifted band was detected in the EMSA assay when the biotin-tagged probe was mixed with SfSoxC protein (Figure 5B, lane 4). This band was not detected when the biotin-tagged mutated probe and SfSoxC protein were used in the assay (Figure 5B, lane 8). Competition assays showed that the binding of SfSoxC to the *SfEH* promoter is specific (Figure 5B, lane 5 and 6). These results show direct binding of SfSoxC protein to the SoxC binding motif in *SfEH* gene.

### *SfSoxC* Activates the Expression of *SfEH*

**FIGURE 6:**
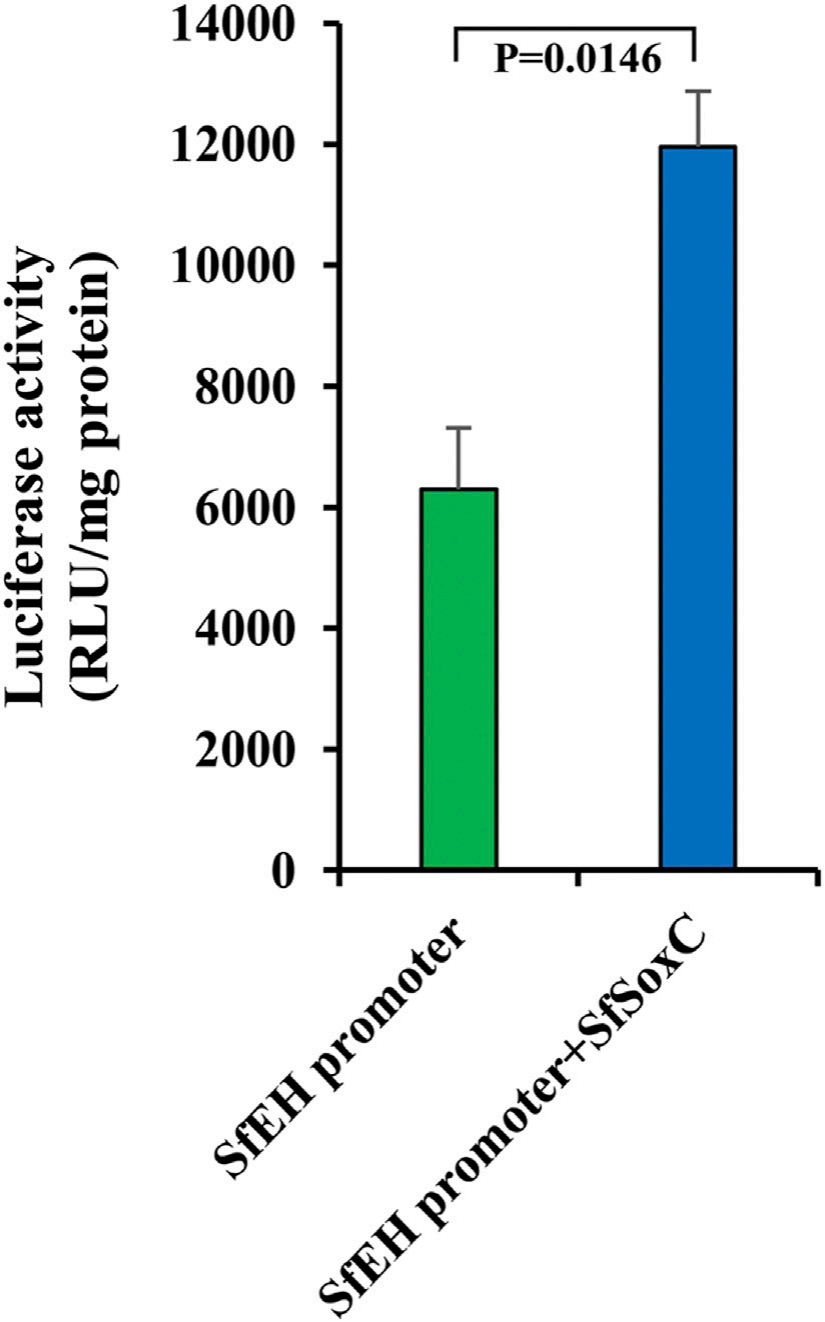
The *SfSoxC* activates the expression of *SfEH*. Luciferase reporter assays in Sf9 cells. Sf9 cells were co-transfected with a reporter construct (*SfEH/P2574-pG5luc*) containing the *SfEH* promoter region (from −2574 to the start codon) and an expression construct (*pIEx-4-SfSoxC*) containing the ORF of *SfSoxC*. The cells were harvested at hr after transfection and the luciferase activity was determined. Means ± SE of three independent experiments are shown.

The *SfSoxC* gene is a transcription factor. To confirm that *SfSoxC* directly activate the expression of *SfEH*, we performed the luciferase reporter assays in Sf9 cells. The activity of luciferase gene expressed under the control of *SfEH* promoter increased in cells overexpressing SfSoxC, suggesting that SoxC binds to its target site in the *EH* promoter and activates transcription (Figure 6).

## Discussion

The ecdysis behavioral sequence is mainly controlled by four critical neuropeptides: EH, ETH, CCAP and bursicon. The data reported here clearly showed that *SoxC* gene is required for adult eclosion. Our recorded video clearly showed that the *T. castaneum* pharate adult could not shed the pupal cuticle completely after the *SoxC* knockdown (Supplementary Video S1). In *M. sexta*, *EH* has been found to have functions such as regulating the extensibility of the latent adult wing and the release of the hormone bursicon (Hewes and Truman, 1991). After ds*EH* was injected into larvae, malformed and impaired wings were observed in *Heortia vitessoides* (Lyu et al., 2019; Li et al., 2020). These observations showed that the lack of *EH* leads to failure in ecdysis. Other reports showed that bursicon regulates adult cuticle tanning and wing expansion after adult emergence in *D. melanogaster*, *B. mori* and *M. sexta* (Baker and Truman, 2002; Huang et al., 2007; Dai et al., 2008). Also, in *T. castaneum*, signaling by CCAP and bursicon is required to modulate the strength of pre-ecdysial behaviors during adult eclosion and to ensure proper postecdysial behavior and wing expansion (Arakane et al., 2008). We observed similar phenotypes in *SoxC* gene knockdown in *T. castaneum* and its knockout in *S. frugiperda*, respectively. We propose that the *SoxC* gene is a critical effector that acts upstream of the neuropeptide genes.

The *SoxC* gene is a transcription factor and a 20E primary-response gene. Some neuropeptide genes are also regulated by 20E (Morton and Truman, 1988; Li et al., 2020). After 20E treatment, the relative expression levels of *EH* and *ETHp* significantly increased (Hiruma and Riddiford, 1986; Li et al., 2020). Our data from *in vivo* (insects) experiments showed that *SoxC* gene and *EH*, *CCAP*, *Bur* genes are upregulated after 20E treatment. Interestingly, when the *SoxC* was knockdown by RNAi, the expression of *EH*, *CCAP*, *Bur* genes decreased significantly. Previous studies showed that EH released into the CNS leads to the release of CCAP and bursicon (Kingan et al., 2001). We hypothesize that the *SoxC* gene regulates expression of *EH* gene. Our EMSA results showed that the SoxC protein directly binds to the SoxC binding motif [5′-AACAATG-3′] present in the promoter of the *EH* gene. The luciferase activity assay also showed that the SoxC protein induces the activity of *EH* gene promoter. Thus, our results demonstrated that *SoxC* transcription factor is a direct regulator of *EH* gene. This is the first report showing that *SoxC* is a key regulator in 20E-induced insect eclosion behavior.

The inseparable relationship between *EH* and *ETH* is complicated. EH causes ecdysis by acting on abdominal neurospheres, which directly or indirectly triggers the release of ETH (White and Ewer, 2014; Nassel and Zandawala, 2019). Our data showed that, in *T. castaneum* treated with SEA, *TcEH* expression increased significantly without any significant change in *TcETHp* expression (Figure 1; Supplementary Figure S1). Interestingly, in *SfSoxC* knockout *S. frugiperda*, the expression of *SfEH* decreased significantly whereas the expression of *SfETHp* increased significantly (Supplementary Figure S3). Some studies indicated that ETH usually ushers in the pre-ecdysis stage, and the EH released into CNS leads to the release of CCAP and bursicon (Kingan et al., 2001). Other reports showed that EH is absolutely needed for ETH release (McNabb et al., 1997; Li et al., 2020). Definitely, there is positive feedback between EH and ETH (Chang et al., 2009; White and Ewer, 2014; Nassel and Zandawala, 2019; Li et al., 2020). While it is still unclear which neuropeptide is an initiator, some reports suggest that the release of ETH is triggered by corazonin thus initiating the EH-ETH feedback loop. EH-associated positive feedback induces a massive release of ETH to initiate ecdysis motor patterns (Kim et al., 2004; Kim et al., 2006). Besides, ETH, which causes only pre-ecdysis in *M. sexta*, can trigger the entire ecdysis sequence in *B. mori* (White and Ewer, 2014). More studies are needed to decipher the functions and relationship of EH and ETH.

Insect wing development and expansion are associated with programmed cell death (Johnson and Milner, 1987; Kimura et al., 2004; Kiger et al., 2007; Natzle et al., 2008). This process is controlled by changes in the levels of 20E, a regulator of the expression of pro-death genes, *reaper* (*rpr*) and *head involution defective* (*hid*), which then repress the inhibitor of apoptosis proteins (IAPs) in *D*. *melanogaster* (Jiang et al., 1997). Studies in *l(2)mbn* cell line and *D*. *melanogaster* insect indicated that the *SoxC* has a pro-death role and acts upstream of the two apoptosis effectors, *rpr* and *dronc* (Chittaranjan et al., 2009). Meanwhile, many 20E-regulated genes require *SoxC* for their proper expression during development. That’s why there are similar patterns of expression in both *EcR* knockdown and *SoxC* mutant fruit flies (Ritter and Beckstead, 2010). In *D*. *melanogaster*, bursicon has already been demonstrated to bind to the bursicon receptor DLGR2 and activate cAMP/PKA signaling. It was proposed that bursicon activation of the cAMP/PKA signaling pathway is likely required for transduction of the hormonal signal that induces wing epidermal cell death after eclosion (Kimura et al., 2004). Our data showed that bursicon expression decreased significantly after *SoxC* knockdown in the red flour beetle and *SoxC* knockout in the fall armyworm. The relationship between *bursicon* and *SoxC* gene requires further studies. We found two putative SoxC binding motifs (5′-TACAATG-3′ and 5′-ATCAAAG-3′) within the promoter region of *bursicon* gene in *S. frugiperda*. Our EMSA data showed that there is no binding between SfSoxC protein and this putative motif (data not shown). Thus, more work is needed to uncover the relationship between *bursicon* and *SoxC*.

The expression of *EH* can be induced by 20E in insects. How 20E regulates the *EH* expression is always unclear. Our data showed that *SoxC* and *EH* are in the involvement of 20E signaling pathway of insect eclosion. And, the present study fills a knowledge gap that *SoxC* gene is a critical effector between 20E and *EH* of insect eclosion process. *SoxC* modulates 20E signaling to promote pupal-adult metamorphosis in insects.

## Data Availability

The datasets presented in this study can be found in online repositories. The names of the repository/repositories and accession number(s) can be found in the article/Supplementary Material.

## Supplementary Material

Supplementary Video S1 The recorded video of eclosion behavior of *T. castaneum* after *TcSoxC* RNAi. The video played at 64x fast forward speed.

Supplementary Figure S1
*SoxC* affects the expression of neuropeptide genes in *T. castaneum* insect. Newly molted final instar larvae of the red flour beetle were injected with 400 ng of ds*SoxC*. 2 µL of SEA solution (dissolved in acetone) was added on the dorsal side abdomen of insects upon entry into quiescent stage and emergence of compound eyes. The control group was treated with acetone. 12 h after treatment, insect total RNA was isolated and quantified with RT-qPCR. **(A)** When treated with SEA, *TcETHp* expression did not change significantly in control larvae but increased significantly in ds*SoxC* larvae. **(B,C)** When treated with SEA, the expression levels of *TcCCAP* and *TcBur* increased significantly in control group but decreased significantly in ds*SoxC* larvae. Means ± SE of three independent experiments are shown (*p* values come from *t test*).

Supplementary Figure S2 The expression of neuropeptide genes after *TcSoxC* knockdown in *T. castaneum* After ds*SoxC* injection, the expression levels of neuropeptide genes *TcEH* **(A)**, *TcETHp* **(B)**, *TcCCAP* **(C)** and *TcBur* **(D)** were determined by RT-qPCR. Means ± SE of three independent experiments are shown (*p* values come from *t tests*). PP: pre-pupa, PD0: newly molted pupa (0 h after ecdysis into the pupal stage), PD1: 24 h after ecdysis into the pupal stage, PD2: 48 h after ecdysis into the pupal stage, PD3: 72 h after ecdysis into the pupal stage, PD4: 96 h after ecdysis into the pupal stage, PD5: 120 h after ecdysis into the pupal stage, AD0: newly eclosed adult.

Supplementary Figure S3 The expression of neuropeptide genes after *SfSoxC* knockout in *S. frugiperda* In the fall armyworm, the expression of neuropeptide genes was determined by RT-qPCR after *SfSoxC* knockout using CRISPR/Cas9. **(A)** *SfEH* was significantly down-regulated with *SfSoxC* knockout at all four stages checked. **(B)** *SfETHp* was significantly up-regulated with *SfSoxC* knockout at all four stages checked. **(C)** *SfCCAP* was significantly down-regulated with *SfSoxC* knockout at all check-points except for PD0 (*p* = 0.02228). **(D)** *Sf-Bur* was significantly down-regulated with *SfSoxC* knockout at all four stages checked. Means ± SE of three independent experiments are shown (*p* values comes from *t tests*). PP: pre-pupa, PD0: newly molted pupa (0 h after ecdysis into the pupal stage), PD7: 7 days after ecdysis into the pupal stage, AD0: newly eclosed adult. WT represent the wildtype insects. *SfSoxC* KO represents the *SfSoxC* gene knockout insects.
