# Supplementary material for: SoxC is Required for Ecdysteroid Induction of Neuropeptide Genes During Insect Eclosion
Source: Front Genet. 2022 Jul 11;13:942884. doi: 10.3389/fgene.2022.942884 (PMC9309532; doi:10.3389/fgene.2022.942884)
Supplement: Supplementary file 1 [file Table1.DOCX]

Table S1 List of primers used in this study

| **Primers** | **Sequence (5’-3’)** | **Notes** |
| --- | --- | --- |
| RT-TcSOXC-F | ATGGTGCCCCAACAAAACATGGATTCA | *TcSoxC* cloning |
| RT-TcSOXC-R | TCAACAGTTGAACACGCCGTCGCTCCA |  |
| RT-SfSOXC-F | ATGGTGCCCCAACAAGTGTCGGATGT | *SfSoxC* cloning |
| RT-SfSOXC-R | CTAAGACGACGTCAGATACGATGAGA |  |
| dsTcSOXC-F | TAATACGACTCACTATAGGGCCCCAAATCGGCGACTTCCTCCAAG | dsRNA synthesis |
| dsTcSOXC-R | TAATACGACTCACTATAGGGTGATCTCGATGTCCGCCAGCTCCTG |  |
| qTcRP49-F | TGACCGTTATGGCAAACTCA | reference gene for RT-qPCR in *T. castaneum* |
| qTcRP49-R | TAGCATGTGCTTCGTTTTGG |  |
| qTcSOXC-F | GTATCCAGATTACAAGTACCGTCC | RT-qPCR for *TcSoxC* |
| qTcSOXC-R | TCCAGCCTCTCTTTCAACTTC |  |
| qTcEH-F | CTACTCTTCGCTTCTTCGCT | RT-qPCR for *TcEH* |
| qTcEH-R | AGTGATGTCCTCGCAATCTG |  |
| qTcETHp-F | TCGTAGTAACACCAACAAGAACAC | RT-qPCR for *TcETHp* |
| qTcETHp-R | TCATACTCAAACCGATCCGCT |  |
| qTcCCAP-F | TTCGTAATCTGCATCTTCGCC | RT-qPCR for *TcCCAP* |
| qTcCCAP-R | CTTCCGACATGATCTGCCTC |  |
| qTcBur-F | TATGTGCTGTCAGGAGTCGG | RT-qPCR for *TcBur* |
| qTcBur-R | GAGAGTGAGACTTGAGGAAATGG |  |
| SfSOXC-sgR-T1-F | TAATACGACTCACTATAGGGGCCTGTACTTGTAGTCGGTTTTAGAGCTAGAAATAGCAAGTTAAAATAA | sgRNA synthesis |
| SfSOXC-sgR-T2-F | TAATACGACTCACTATAGGCAACACCCCTGTTGTTATGTTTTAGAGCTAGAAATAGCAAGTTAAAATAA | sgRNA synthesis |
| SfSOXC-sgR-T3-F | TAATACGACTCACTATAGGTCTACTTCGCAGTCTGCAGTTTTAGAGCTAGAAATAGCAAGTTAAAATAA | sgRNA synthesis |
| gRNA-R | AAAAGCACCGACTCGGTGCCACTTTTTCAAGTTGATAACGGACTAGCCTTATTTTAACTTGCTATTTC | sgRNA synthesis |
| SfSOXC-KO-F | CACCTTGCATACTAATTGGACCTG | Knockout mutation analysis |
| SfSOXC-KO-R | AACTCAAAGTGGGACCCTGATG |  |
| qSfGAPDH-F | GTGCCCAGCAGAACATCAT | reference gene for RT-qPCR in *S. frugiperda* |
| qSfGAPDH-R | GGAACACGGAAAGCCATAC |  |
| qSfSOXC-F | GCGGAAATATCCAAGAACCTC | RT-qPCR for *SfSoxC* |
| qSfSOXC-R | CACTGGGTACTGATGGAACTG |  |
| qSfEH-F | CGCTGCTTTCTTCATCTTCGT | RT-qPCR for *SfEH* |
| qSfEH-R | CTTTCCCTTGAAGGTGATGCAG |  |
| qSfETHp-F | AGTACTATTGGCATACTGCCT | RT-qPCR for *SfETHp* |
| qSfETHp-R | AAGGAAGCTGATAGAATTTGGG |  |
| qSfCCAP-F | GAGTGTTGCCATACAGCCTC | RT-qPCR for *SfCCAP* |
| qSfCCAP-R | GCCTGGATAGCTCTTCAATGG |  |
| qSfBur-F | GGATGTATTCCTAAAGCGATACC | RT-qPCR for *SfBur* |
| qSfBur-R | CTTCTTCAGCGTATCCAGCA |  |
| SfEH-probe-F | TGTCGATTCAAAGGTAATT | SfSoxC binding motif within *SfEH* for EMSA probe |
| SfEH-probe-FM | TGTCGAGGACGCTGTAATT | Mutated SfSoxC binding motif within *SfEH* for EMSA probe |
| SfEH-KpnITY-F | AGAACATTTCTCTATCGATAGGTACCATCAATCACGACATTTCGTATCC | SfEH/P2547-pG5luc vector construction |
| SfEH-HindIIITY-R | CCAACAGTACCGGAATGCCAAGCTTTGATCTGGGAAAATGTCAACGT |  |
| SfSOXC-NcoITY-F | GATCGTGAACAACCAAGTGACCATGGTGCCCCAACAAGTGTCGGATG | pIEx-4-SfSOXC vector construction |
| SfSOXC-AscITY-R | ACCGGTACCGTCGACCTGCAGGCTAAGACGACGTCAGATACGATGAG |  |
