# Supplementary figures and images for: SoxC is Required for Ecdysteroid Induction of Neuropeptide Genes During Insect Eclosion
Source: Front Genet. 2022 Jul 11;13:942884. doi: 10.3389/fgene.2022.942884 (PMC9309532; doi:10.3389/fgene.2022.942884)

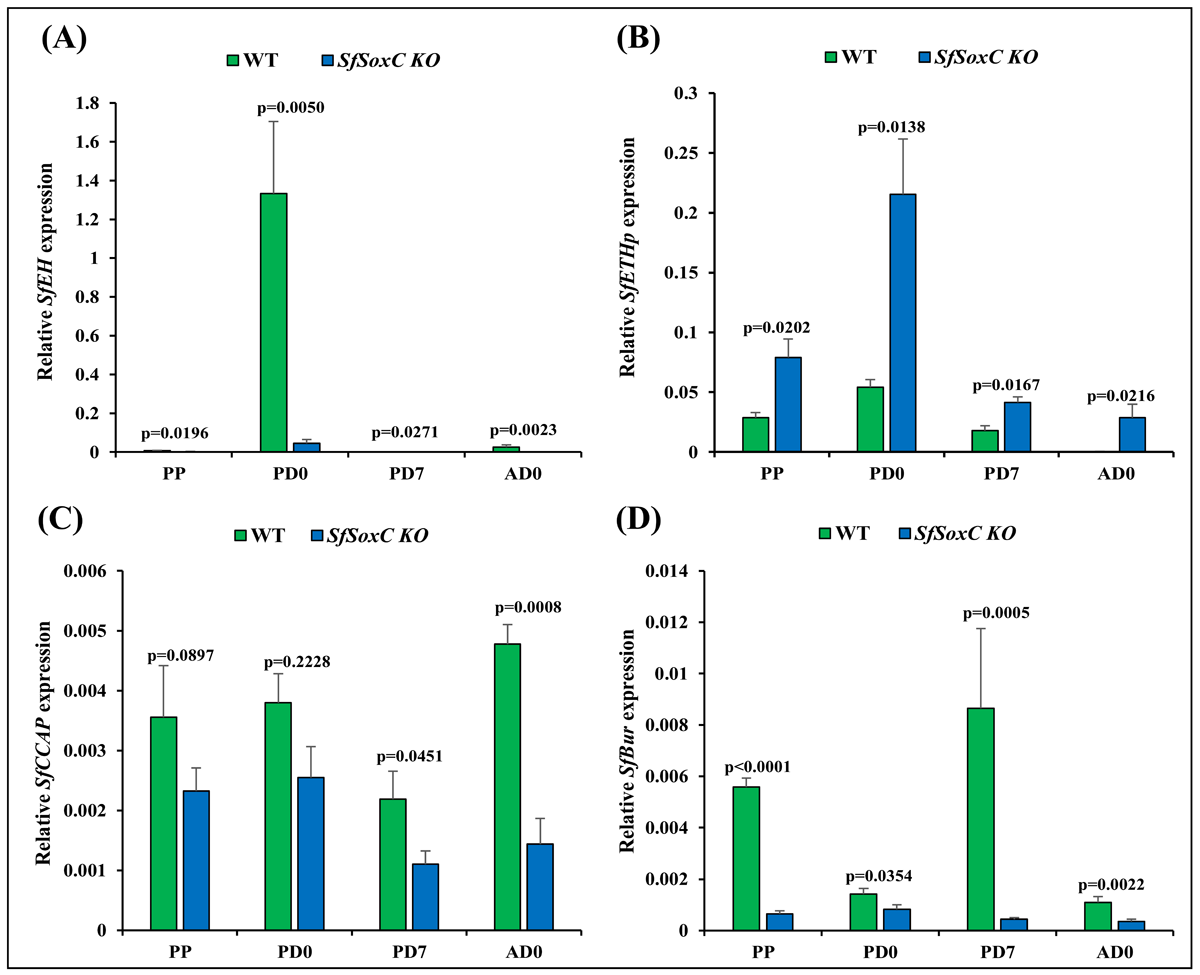

Supplement: Supplementary file 2 [file Image3.TIF]

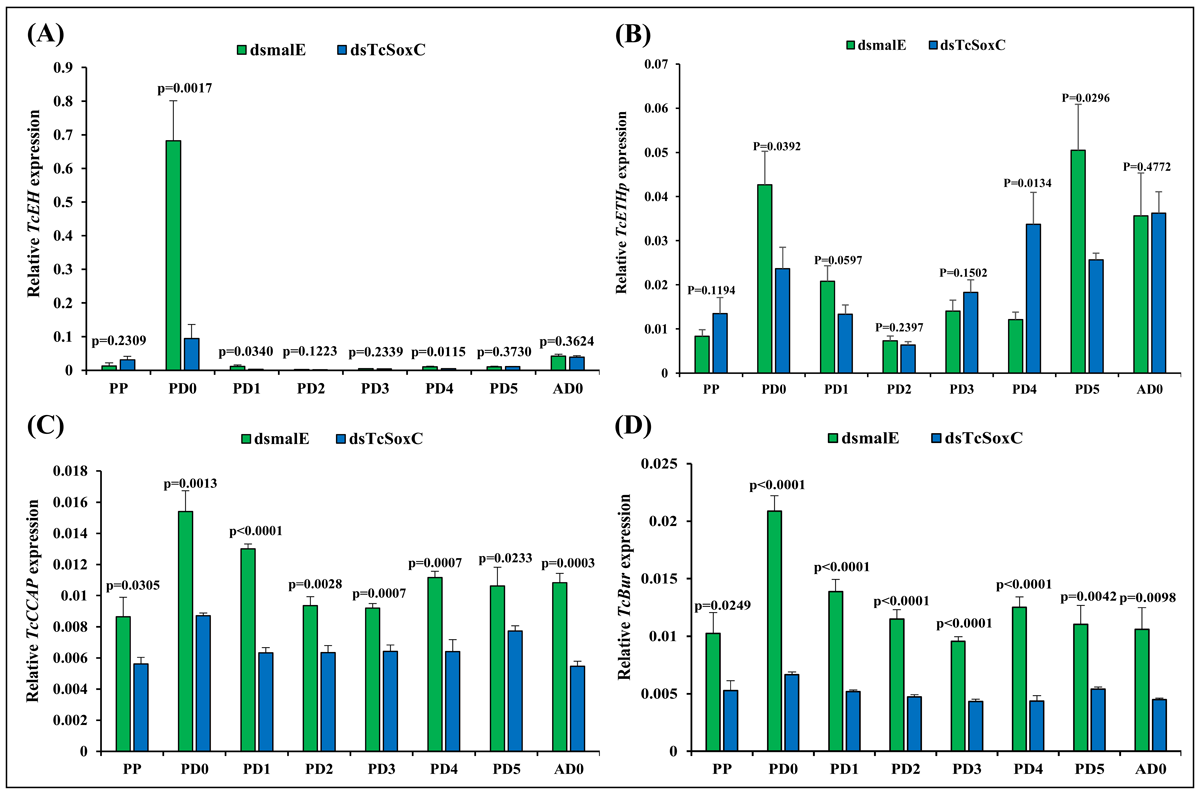

Supplement: Supplementary file 3 [file Image2.TIF]

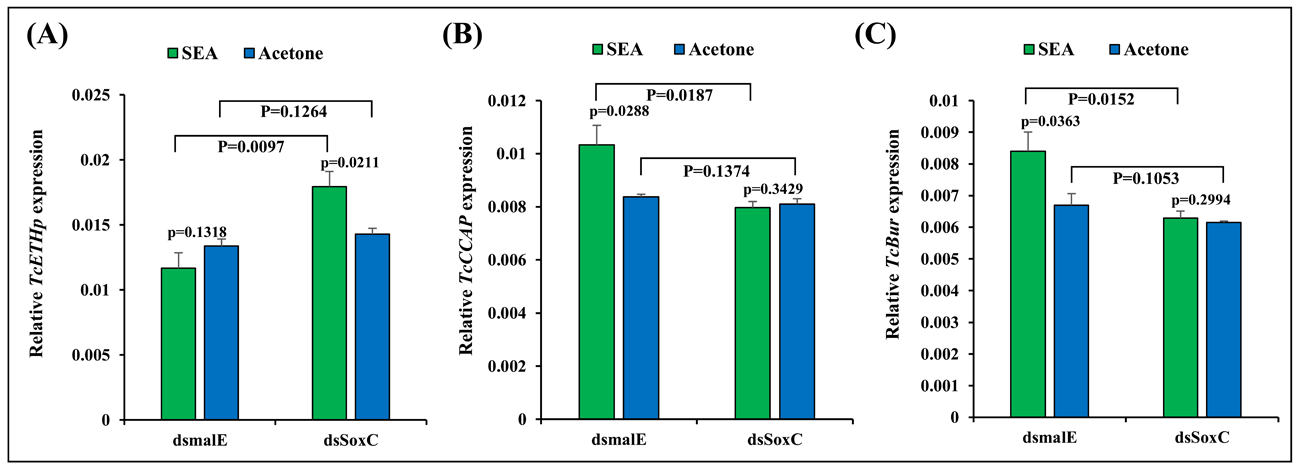

Supplement: Supplementary file 4 [file Image1.TIF]
