# Supplementary material for: SoxC is Required for Ecdysteroid Induction of Neuropeptide Genes During Insect Eclosion
Source: Front Genet. 2022 Jul 11;13:942884. doi: 10.3389/fgene.2022.942884 (PMC9309532; doi:10.3389/fgene.2022.942884)
Supplement: Supplementary file 5 [file Table2.DOCX]

Table S2. The CRISPR/Cas9 system induced mutagenesis in the fall armyworm

| **Target gene** | **Injected components** | **NO. of eggs injected** | **NO. of eggs hatched** | **Mutation rate** | **No. of dead 1^st^ instar larvae** | **No. of dead pupae** | **NO. of eclosion abnormal adults** |
| --- | --- | --- | --- | --- | --- | --- | --- |
| *SfSoxC* | Cas9 + sgRNA target A + sgRNA target B + sgRNA target C | 216 | 169 | 83.33%  (25/30) | 39 | 39 | 48 |
